# Potassium and zinc improves physiological performance, nutrient use efficiency, and productivity of wheat

**DOI:** 10.3389/fpls.2024.1363248

**Published:** 2024-07-11

**Authors:** Abeer Nawaz, Muhammad Amjad Bashir, Wazir Ahmed, Ijaz Ahmad, Abdur Rehim, Rao Muhammad Ikram, Syed Shahid Hussain Shah, Muhammad Yasir Khurshid, Munir Jamil Rusan, Rashid Lubani, Shahzad Saleem, Tanveer Ul Haq, Muhammad Asif Ali

**Affiliations:** ^1^ Department of Soil and Environmental Sciences, Muhammad Nawaz Shareef University of Agriculture, Multan, Pakistan; ^2^ Sichuan Provincial Key Laboratory of Philosophy and Social Sciences for Monitoring and Evaluation of Rural Land Utilization, Chengdu Normal University, Chengdu, China; ^3^ Engro Fertilizers Limited, Lahore, Pakistan; ^4^ Department of Soil Science, FAS&T, Bahauddin Zakariya University, Multan, Pakistan; ^5^ Department of Agronomy, Muhammad Nawaz Shareef University of Agriculture, Multan, Pakistan; ^6^ Department of Natural Resources and Environment, Jordan University of Science and Technology, Al Ramtha, Irbid, Jordan; ^7^ Arab Potash Company, Al Ramtha, Irbid, Jordan; ^8^ Agri Development, United Agro Chemicals, Lahore, Pakistan

**Keywords:** fertilizer use efficiency, nutrition, photosynthesis, K, Zn

## Abstract

Despite the critical role of balanced nutrition in crop productivity, the use of potash (K) and zinc (Zn) is not much practiced by Pakistani farmers. The reduced nutrient uptake and crop productivity together increase the costs associated with fertilization and revisit farmers’ confidence in the efficacy and profitability of fertilizers. To address this problem, a field study was conducted in the research area of the MNS-University of Agriculture, Multan, in collaboration with Engro Fertilizers Limited. The research plan consisted of five treatments, including T_1_ = control (without N, P, K, and Zn fertilizers), T_2_ = NP in practice (NP at 32–23–0 kg acre^-1^), T_3_ = recommended NP (NP at 48–34.5 kg acre^-1^), T_4_ = balanced NPK (NP+K at 48–34.5–30 kg acre^-1^), and T_5_ = balanced NPK + Zn (NPK+Zn at 48–34.5–30 + 7.5 kg acre^-1^). Wheat was used as a test crop, and its growth, yield, and physiological and nutritional parameters were studied. The results indicated that NPK+Zn balanced nutrition increased plant height, spike length, photosynthetic rate, water use efficiency, transpiration rate, stomatal conductance, and grain yield by 13%, 15%, 44%, 60%, 63%, 39%, and 78%, respectively, compared with the control. It was found that the combined application of NP, K, and Zn improved the recovery efficiency of applied nutrients, i.e., nitrogen recovery efficiency (NRE) by 230%, phosphorus recovery efficiency (PRE) by 136%, potassium recovery efficiency (KRE) by 135%, and zinc recovery efficiency (ZnRE) by 136% compared to NP-alone application. Agronomic use efficiency of applied fertilizers, such as potassium agronomic use efficiency (KAUE) by 71%, phosphorus agronomic use efficiency (PAUE) by 72%, nitrogen agronomic use efficiency (NAUE) by 70%, and zinc agronomic use efficiency (ZnAUE) by 72%, was observed compared to NP-alone application. The results showed that NPUE, PPUE, NPUE, and ZnPUE were reduced by 5%, 3%, 3%, and 5%, respectively, compared to NP-alone application. Our findings suggest that K and Zn should be made an essential part of wheat nutrition management for higher yield and better quality of produce.

## 1 Introduction

Wheat is the “king of the world cereals,” being the staple food of ~35% of the global population. Pakistan produces 27.5 million metric tons of wheat against the requirement of 29.1 million metric tons. The average yield per hectare is still 20% lower than the wheat average yield per hectare in India (Food and Agriculture Organization (FAO), 2019). Conventional methods of crop cultivation, late sowing, improper weed management, limited supply of water at critical growth stages, and non-judicious application of fertilizers, particularly K fertilizers, along with some other conflicts decrease wheat production, which is a serious concern.

Potassium (K) is the third most essential macronutrient that plays an important role in plant growth, metabolism, and stabilizing crop yield (Huang et al., 2022). It also activates more than 80 enzymes and is involved in various physiological processes, including respiration, photosynthesis, protein synthesis, translocation of photo-assimilates, and stomatal regulation (Perelman et al., 2022). Its deficiency directly involves chlorophyll degradation, enhanced production of reactive oxygen species (ROS), and reduction in photosynthetic activity (Hafsi et al., 2014). Thus, an adequate supply of K is essential for strong stalks, plump grains in cereals, kernel weight, protein, and amino acid contents (Zewdie and Reta, 2021). It also increases the size and improves the color and flavor of fruits and tubers. Furthermore, it extends the shelf life by increasing the resistance to several injuries during transportation and storage (Wakeel and Magen, 2017). Potassic supply increases tolerance against abiotic stresses (Mostofa et al., 2022) and improves drought tolerance in crops by improving the osmotic adjustment (da Silva et al., 2021). In addition, K is an important nutrient for numerous roles in plants such as protein synthesis, enzymatic activity, resistance against stresses, stomatal regulation, and xylem/phloem transportation. Although Pakistani soils are moderate in K concentration, due to improper cultivation practices, K deficiency might disturb crop performance (Raza et al., 2021).

Zinc (Zn) is also a key micronutrient, and its adequate supply improves cereal productivity (Butail et al., 2022). It improves the flour quality of wheat (Ali Khan et al., 2018). It is the first most deficient micronutrient reported in the soils of Pakistan, and almost all soils of Pakistan are Zn deficient (Yaseen et al., 2013). It also plays significant roles in enzymatic reactions essential for photosynthesis, improves the quality of food products, and enhances per unit area production (Ehsanullah et al., 2015). Zn acts as co-factor for 300 enzymes in plants and is considered an essential nutrient for plant growth. Due to less Zn availability due to its fixation with clay particles, high pH range, and soil calcareousness, Pakistani soils are also deficient in Zn (Raza et al., 2021).

To meet food security challenges, the upsurge in demand for chemical fertilizers was observed after the Green Revolution in 1960. With a gradual rise in the use of nitrogenous and phosphatic fertilizers for crops, due to the imbalanced use of fertilizers, the corresponding rise in crop yields was not achieved (Penuelas et al., 2023). K is becoming deficient with every year due to intensive farming and lesser use of potassic fertilizer in Pakistan (Wakeel et al., 2022). The challenge for sustainable crop production is now to introduce strategies in cropping systems such as integration of multiple nutrients that could increase nutrient use efficiency.

In this context, by considering the importance of K and Zn fertilization, the present study was aimed to evaluate the comparative effects of K and Zn use with recommended and conventional practices on the physiological and agronomic performance of wheat crops. This study was a significant approach toward the effective and efficient use of mineral fertilizers with an understanding of the integrative use of plant essential nutrients. The study hypothesizes that the application of K and Zn could improve wheat crop performance and yield.

## 2 Materials and methods

### 2.1 Experiment site and treatment plan

A field trial was conducted at the research farm of the MNS-University of Agriculture, Multan in collaboration with Engro Fertilizers Limited to investigate the balanced use of fertilizers along with K application for yield and quality production of wheat on calcareous soil. Soil sample at 0–15-cm depth before the experiment was collected to measure the soil physicochemical properties (**Table 1**). The climate of the experimental area is semi-arid, with warm summers and mild winters, and the mean annual temperature and precipitation are about 25.6°C and 186 mm, respectively. The study was laid down in an RCBD design with five treatments and three replicates. The treatments were T_1_ = control (without N, P, K, and Zn fertilizers), T_2_ = NP in practice (NP at 32–23–0 kg acre^-1^), T_3_ = recommended NP (NP at 48–34.5 kg acre^-1^), T_4_ = balanced NPK (NP+K at 48–34.5–30 kg acre^-1^), and T_5_ = balanced NPK + Zn (NPK+Zn at 48–34.5–30 + 7.5 kg acre^-1^).

**Table 1:** Effect of treatment on yield parameters.

Treatment	Plant height (cm)	Productive tiller plant ^-1^	Spike length (cm)	Spikelet per spike	Grain per spike
Control	89.28 ± 0.26e	14.14 ± 0.6	11.03 ± 0.71	12.32 ± 0.01d	23.86 ± 0.03e
Farmer practice	91.35 ± 0.05d	15.11 ± 0.6	13.89 ± 0.02	16.27 ± 0.01c	44.71 ± 0.44d
Recommended NP	101.23 ± 0.21c	15.67 ± 0.6	15.42 ± 0.10	16.27 ± 0.01c	48.44 ± 0.08c
NPK	101.82 ± 0.62b	16.11 ± 0.6	15.62 ± 0.02	17.37 ± 0.02b	50.33 ± 0.01b
NPK+Zn	103.15 ± 0.05a	17.18 ± 0.6	15.97 ± 0.04	17.46 ± 0.03a	50.81 ± 0.03a

The full dose of P and K was applied at sowing, while N was applied in two splits, i.e., 25 days after sowing and 55 days after sowing. Seeds of wheat cv. Faisalabad 2008 were sown in all plots after seedbed preparation and the addition of P and K fertilizers. Herbicides were used to control weeds, while plots were irrigated as per the recommended production technology. At maturity, data related to growth and yield was taken.

### 2.2 Crop data

Morphological data: Data related to growth attributes was taken before harvesting the crop, while data on yield attributes was taken after harvesting the crop.

Physiological data: Data related to different physiological parameters such as transpiration and photosynthetic rate with the help of an infrared gas analyzer (IRGA; Analytical Development Company, Hoddesdon, UK) and chlorophyll contents was collected using Portable Chlorophyll meter (Konica Minolta, Osaka, Japan).

Plant and soil analyses: For plant analysis, composite samples of plant parts from each treatment were taken, dried at 65°C, ground, and used for the determination of N, P, and K contents using standard procedures given by Moodie (1959), Chang and Jackson (1958), Wolf (1982), and Chapman and Pratt (1961).

For soil analysis, N, P, K, and Zn were determined by following the procedures given in the ICARDA manual (Estefan et al., 2013).

### 2.3 Fertilizer uptake and use efficiency

N, P, and K concentrations in shoot and grains of wheat were determined (Wolf, 1982; Jackson, 1960; Chapman and Pratt, 1961). N, P, and K contents (uptake) as a product of concentration and dry weight plant^-1^ and different forms of nutrient use efficiency were also calculated using following formulae by Ahmed et al. (2020) and Lubao et al. (2023).


(1) $$\text{FAUE }\left(\text{kg }{\text{kg}}^{-1}\right)=\frac{{\text{GY}}_{\text{T}}-{\text{GY}}_{\text{C}}}{\text{NA}}$$

(2) $$\text{FAR}\left(\%\right)=\frac{{\text{NU}}_{\text{T}}-{\text{NU}}_{\text{C}}}{\text{NA}}\times 100$$

(3) $$\text{FPUE}\left(\text{kg }{\text{kg}}^{-1}\right)=\frac{{\text{GY}}_{\text{T}}-{\text{GY}}_{\text{C}}}{{\text{NU}}_{\text{T}}-{\text{NU}}_{\text{C}}}$$

(4) $$\text{IFR}\left(\%\right)=\frac{{\text{NU}}_{\text{T}}-{\text{NU}}_{\text{C}}}{{\text{NU}}_{\text{T}}}\times 100$$

(5) $$\text{FACE}\left(\%\right)=\frac{{\text{NU}}_{\text{T}}-{\text{NU}}_{\text{C}}}{\text{NA}}\times 100$$

(6) $$\text{FUTE}\left(\%\right)=\frac{{\text{GY}}_{\text{C}}}{{\text{GY}}_{\text{T}}}\times 100$$

(7) $$\text{FSF}\left(\%\right)=\frac{{\text{GY}}_{\text{T}}-{\text{GY}}_{\text{C}}}{GY_{\text{T}}}\times 100$$

where FAUE = fertilizer agronomic use efficiency, GY_T_ = grain yield in treated plot, GY_c_ = grain yield in control plot (Equation 1), NU_T_ = nutrient uptake in treated plot, NU_c_ = nutrient uptake in control plot, NA = nutrient applied, FAR = fertilizer apparent recovery (Equation 2), FPUE = fertilizer physiological use efficiency (Equation 3), IFR = internal fertilizer remobilization (Equation 4), FACE = fertilizer nutrient acquisition efficiency (Equation 5) FUTE = fertilization efficiency (Equation 6), and FSF = fertilizer stress factor (Equation 7).

### 2.4 Statistical analysis

Analysis of variance (ANOVA) was used to determine the effect of each treatment. When the *F* ratio was significant, multiple mean comparisons were performed using Fisher’s least significance test (5% probability level) using Statistix 9^©^ (Steel et al., 1997).

## 3 Results

### 3.1 Effect of treatments on yield parameters

#### 3.1.1 Plant height (cm)

The data associated with the effect of different combinations of N, P, K, and Zn fertilizers on plant height is given in **Table 1**. K and Zn supplementation showed a positively significant influence on the height of plants. Plant height was increased with the application of K. In comparison to the control, balanced NPK+Zn improved the plant height (15.53%), followed by balanced NPK (14.05%), recommended NP (13.42%), and practiced NP (2.3%) respectively.

#### 3.1.2 Tillers per plant

The soil application of potash and Zn had a positive effect on productive tillers produced in wheat crops. The plants with K and Zn supplements enhanced the tillers’ production than other treatments. Moreover, differences among treatments were also significant. The maximum number of tillers were observed with the application of NPK+Zn (21.50%), followed by balanced NPK36+ (13.93%), recommended NP (10.82%), and practiced NP (6.68%) as compared to the control (**Table 1**).

#### 3.1.3 Spike length (cm)

A similar trend was observed with the spike length (**Table 1**). The spike length was improved with the application of NPK+Zn (41.47%), followed by balanced NPK (41.58%), recommended NP (39.76%), and practiced NP (25.72%) as compared to the control (**Table 1**).

#### 3.1.4 Number of spikelets per spike

The number of spikelets was also improved with the application of K and Zn (**Table 1**). The maximum number of spikelets was observed with NPK+Zn (41.72%), followed by balanced NPK (41.58%), whereas practiced NP and balanced NP showed similar results.

#### 3.1.5 Grains per spike

Grains per spike were also influenced by the application of K and Zn. The grains per spike were improved with the application of NPK+Zn (212.15%), followed by balanced NPK (210.14%), recommended NP (103.02%), and practiced NNP (87.38%), respectively.

#### 3.1.6 1,000-grains weight

Application of NP with and without K and Zn significantly affected 1,000-grains weight (**Table 2**).

**Table 2:** Effect of treatment on yield parameters.

Treatment	1,000-grains weight (grams)	Grains yield (t/ha)	Biological yield (t/ha)
Control	17.72 ± 0.020e	1.44 ± 0.05d	9.31 ± 0.09c
Farmer practice	31.36 ± 0.0170d	2.83 ± 0.05c	12.27 ± 0.01b
Recommended NP	38.16 ± 0.051c	3.81 ± 0.04b	13.15 ± 0.01a
NPK	39.06 ± 0.020b	4.73 ± 0.07a	13.46 ± 0.02a
NPK+Zn	40.03 ± 0.031a	5.03 ± 0.13a	13.54 ± 0.02a

The values herein indicate mean ± SD (n = 3).

The letter shows the statistical differences.

The results demonstrated that balanced fertilizers were more effective than NP-alone application for improving 1,000-grains weight. The highest value was observed with NPK+Zn (125.90%), followed by balanced NPK (120.43%), recommended NP (115.35%), and practiced NP (76.98%), respectively.

#### 3.1.7 Grain yield

The results elaborated that the grain yield could be enhanced with the supplementation of K and Zn. Grain yield was improved with NPK+Zn (248.32%), followed by balanced NPK (227.04%), recommended NP (163.52%), and practiced NP (96.24%) as compared to the control. In comparison with practiced NP, NPK+Zn (77.50%) showed a higher yield, followed by balanced NPK (66.66%) and recommended NP (34.29%).

#### 3.1.8 Biological yield

The results elaborated that the biological yield could be enhanced with the supplementation of K and Zn. Grain yield was improved with NPK+Zn (45.48%), followed by balanced NPK (44.53%), recommended NP (41.23%), and practiced NP (31.74%) as compared to the control.

### 3.2 Effect of treatments on plant physiological parameters

#### 3.2.1 Chlorophyll contents

**Figure 1:**
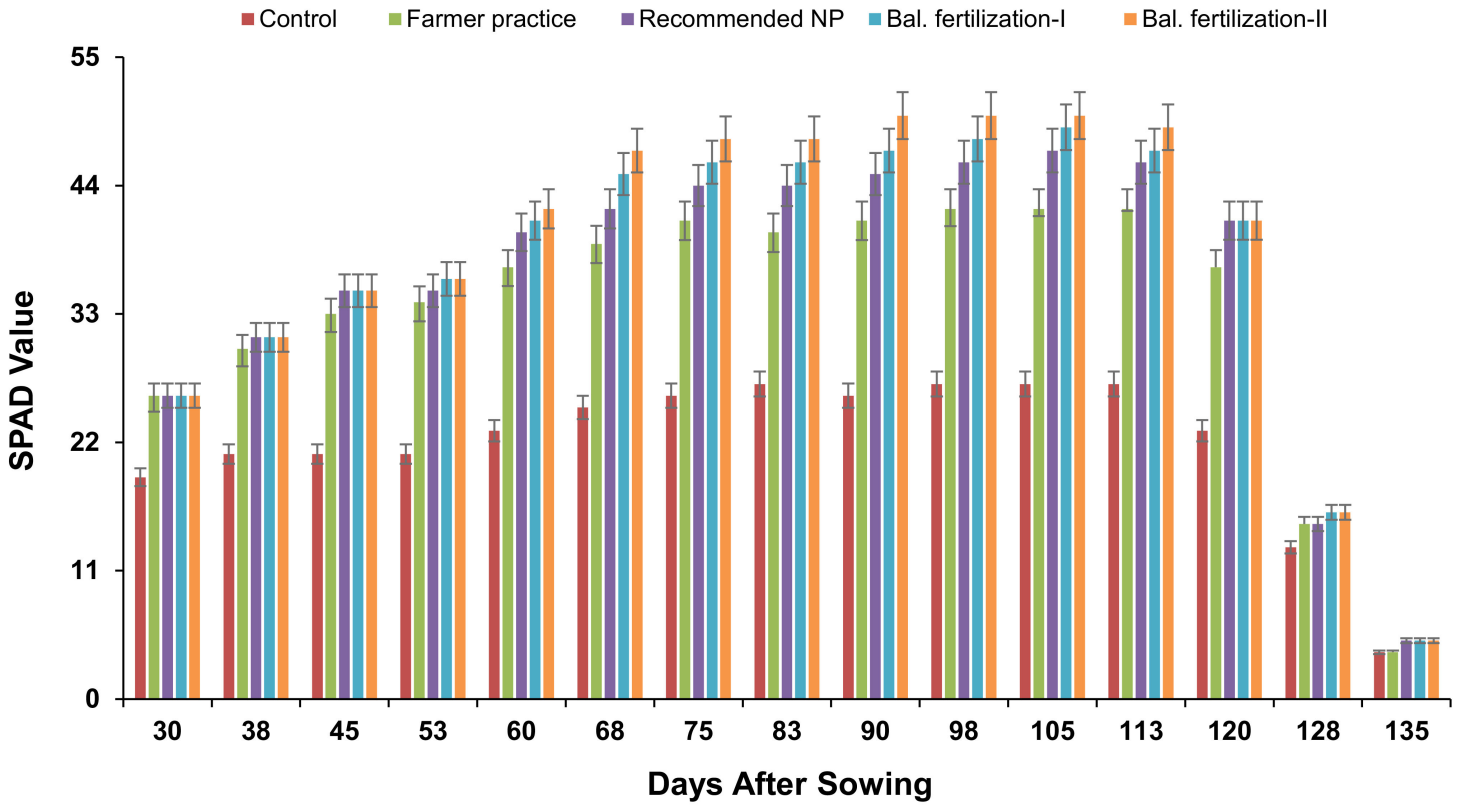
SPAD values as affected by different fertilizer treatments with crop age. The data is presented as mean ± SD.

The data associated with the effect of different combinations of N, P, K, and Zn fertilizers on SPAD chlorophyll contents is given in **Figure 1**. SPAD chlorophyll contents increased with the age of the crop with differential response to the treatments applied. The application of K improved the SPAD contents, which were further enhanced by the application of Zn. The results showed 2% to 3% improvement in chlorophyll contents due to either K (T_4_) or K+Zn (T_5_) application compared to the recommended NP (T_3_).

#### 3.2.2 Photosynthetic rate

**Figure 2:**
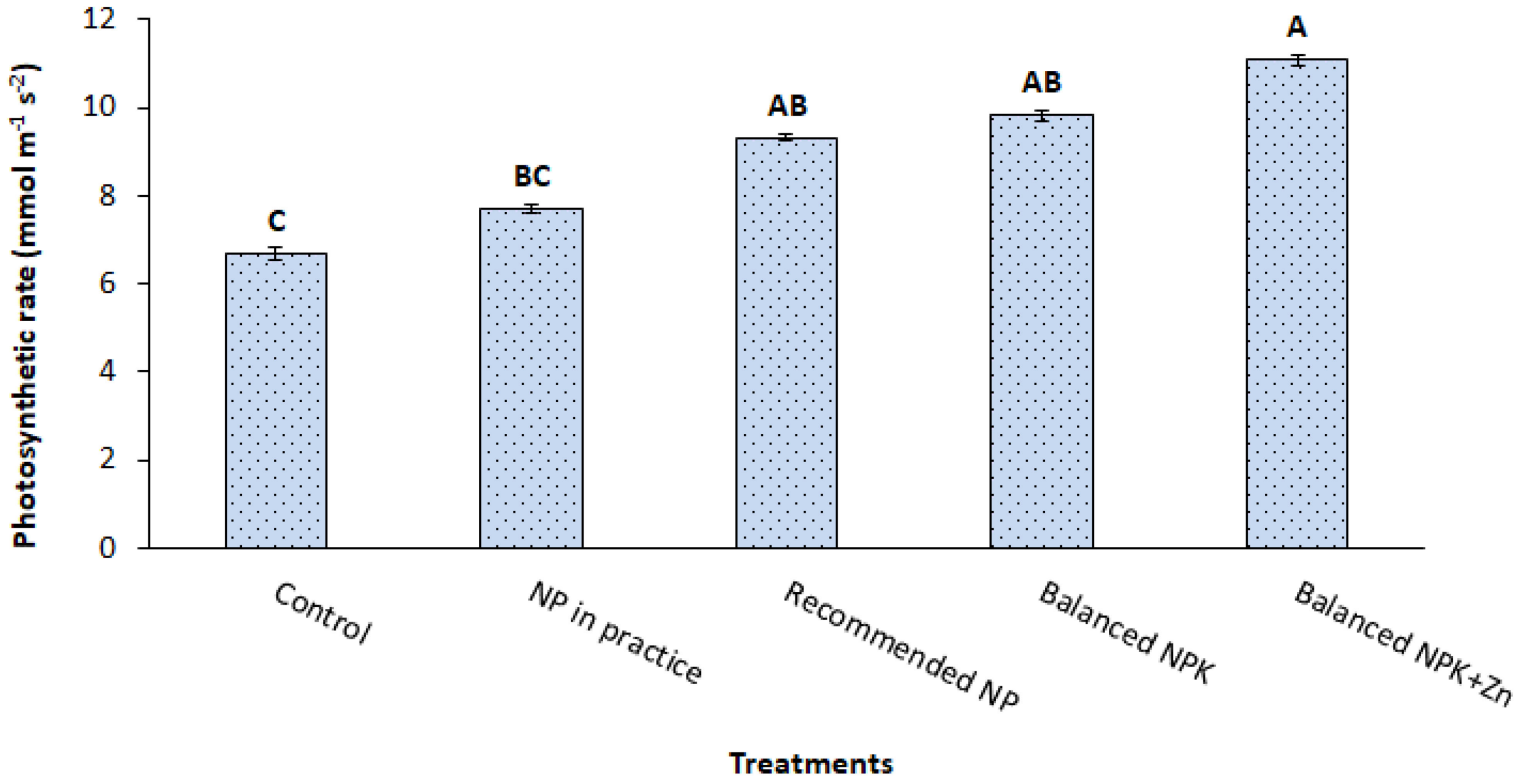
Effect of treatments on photosynthetic rate (mmol m^-1^ s^-2^). The data is presented as mean ± SD. The letter shows the statistical differences.

The data associated with the effect of different combinations of N, P, K, and Zn fertilizers on photosynthetic rate is given in **Figure 2**. The minimum photosynthetic rate (8 mmol m^-2^ s^-1^) on the recommended application of NP (T_3_) was increased up to 11 mmol m^-2^ s^-1^ in the treatment having the application of NP with K+Zn (T_5_). The results showed 5% to 19% increase in photosynthetic rate due to either additional K (T_4_) or K+Zn (T_5_) application compared to the recommended NP (T_3_).

#### 3.2.3 Transpiration rate and stomatal conductance

**Figure 3:**
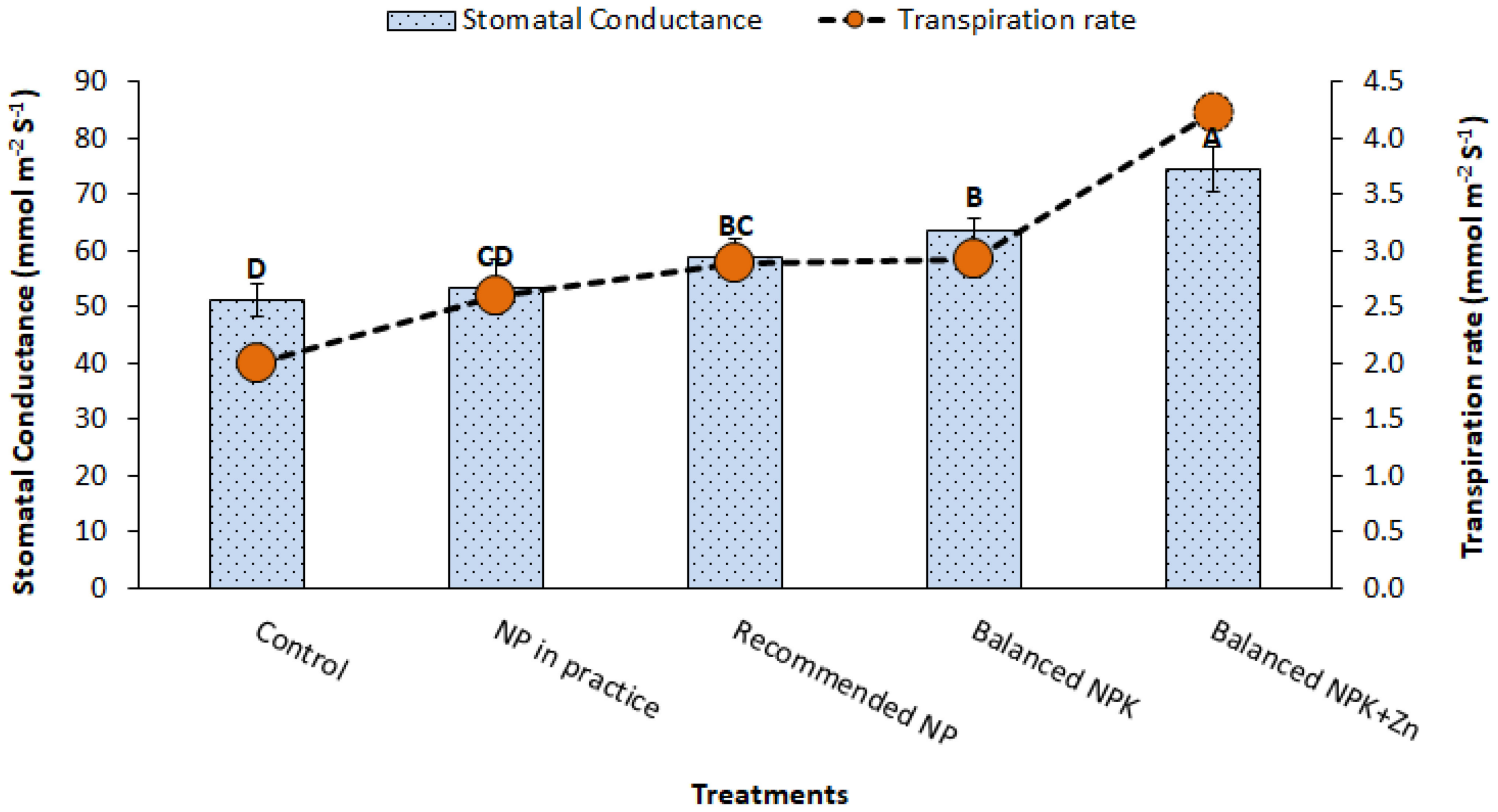
Effect of treatments on stomatal conductance and transpiration rate. The data is presented as mean ± SD. The letter shows the statistical differences.

Transpiration rate (2.6 mmol m^-2^ s^-1^) on the sole application of recommended NP (T_3_) was increased up to 4.2 mmol m^-2^ s^-1^ in treatment T_5_ having recommended NP with K+Zn. Stomatal conductance likewise increased from 59 mmol m^-2^ s^-1^ (T_3_) to 74 mmol m^-2^ s^-1^ (T_5_) (**Figure 3**). The results showed 1%–47% and 8%–26% increase in transpiration rate and stomatal conductance, respectively.

#### 3.2.4 Water use efficiency

**Figure 4:**
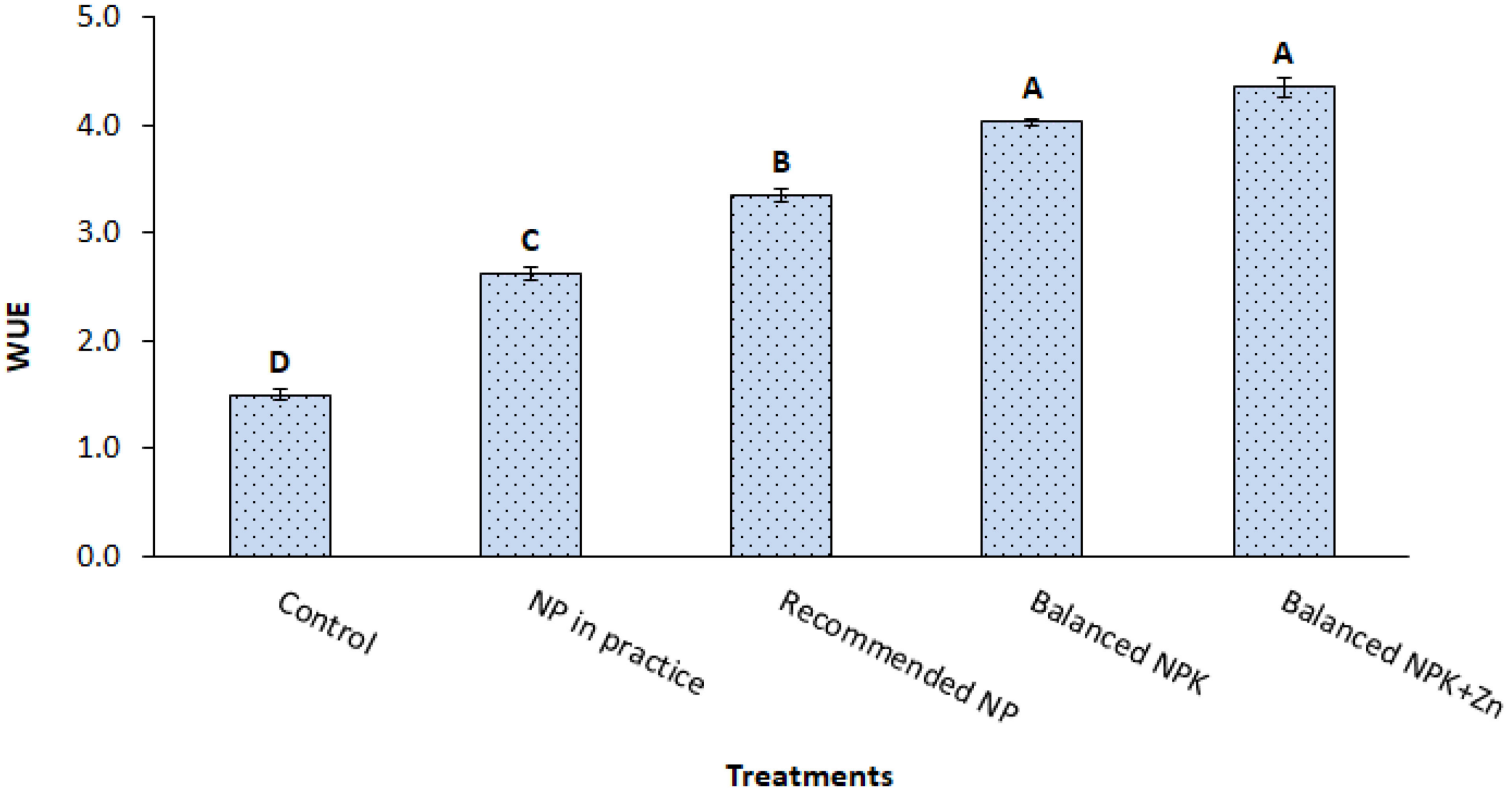
Impact of different fertilizer treatment on water use efficiency. The data is presented as mean ± SD. The letter shows the statistical differences.

The data given in **Figure 4** explores the minimum WUE (2.6) on the application of recommended NP (T_3_) alone, which was increased up to 4.4 in the treatment having the application of NP with K+Zn (T_5_). The results showed 20% and 30% improvement in WUE due to the additional application of either K (T_4_) or K+Zn application (in T_5_) along with the recommended NP application, respectively.

### 3.3 Effect of treatments on grain nutrient content and uptake

Grain nutrient concentration was influenced with the supplementation of K and Zn fertilizers (**Table 3**). The results revealed that grain P content was improved with NPK+Zn (546.27%), followed by balanced NPK (508.96%), recommended NP (485.07%), and practiced NP (261.91%) as compared to the control. Grain K content was also increased with NPK+Zn (29.97%), followed by balanced NPK (24.39%) and practiced NP (9.06%) as compared to the control. Furthermore, grain Zn content was also enhanced with NPK+Zn (46.61%), followed by balanced NPK (34.28%), recommended NP (28.77%), and practiced NP (21.95%) as compared to the control.

**Table 3:** Effect of treatments on grain nutrient content.

Treatment	P (%)	K (%)	Zn (ppm)
Control	0.067 ± 0.008	0.287 ± 0.004	24.33 ± 0.91
Farmer practice	0.242 ± 0.001	0.313 ± 0.003	29.67 ± 0.88
Recommended NP	0.392 ± 0.002	0.303 ± 0.003	31.33 ± 0.78
Bal. fertilization-I	0.408 ± 0.002	0.357 ± 0.002	32.67 ± 0.98
Bal. fertilization-II	0.433 ± 0.004	0.373 ± 0.004	35.67 ± 0.81

The values herein indicate mean ± SD (n = 3).

Nutrient uptake was also improved with the combined application of nutrients (**Table 4**). Grain P uptake was improved with NPK+Zn (2,156.06%), followed by balanced NPK (1,894.70%), recommended NP (1,442.17%), and practiced NP (608.84%) as compared to the control. K uptake was also positively increased with the integration of Zn and K. Grain K uptake was improved with NPK+Zn (167.30%), followed by balanced NPK (162.70%), recommended NP (148.99%), and practiced NP (79.43%) as compared to the control. Grain Zn uptake was also improved with NPK+Zn (410.42%), followed by balanced NPK (338.82%), recommended NP (239.23%), and practiced NP (139.09%) as compared to the control.

**Table 4:** Effect of treatments on grain nutrient uptake.

Treatment	P (kg acre^-1^)	K (kg acre^-1^)	Zn (g acre^-1^)
Control	0.396 ± 0.02	20.645 ± 0.50	14.399 ± 0.95
Farmer practice	2.807 ± 0.02	37.044 ± 0.85	34.426 ± 0.84
Recommended NP	6.107 ± 0.05	51.405 ± 0.92	48.846 ± 0.58
Bal. fertilization-I	7.899 ± 0.09	54.235 ± 0.88	63.185 ± 0.77
Bal. fertilization-II	8.934 ± 0.08	55.184 ± 0.95	73.495 ± 0.69

The values herein indicate mean ± SD (n = 3).

### 3.4 Effect of treatment on nutrient use efficiency

**Figure 5:**
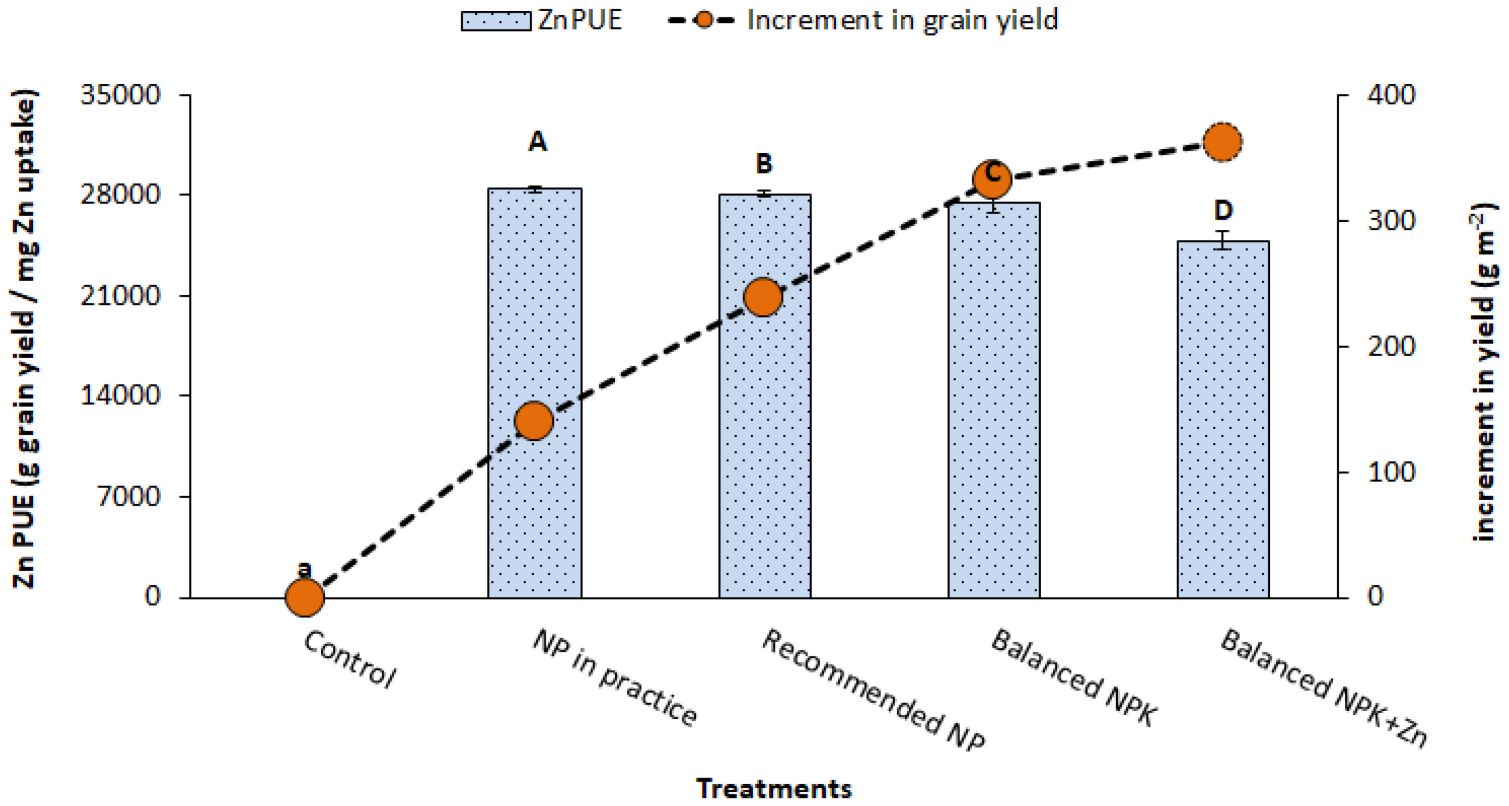
Effect of treatments on ZnPUE and increment in yield (g m^-2^). The data is presented as mean ± SD. The letter shows the statistical differences.

**Figure 6:**
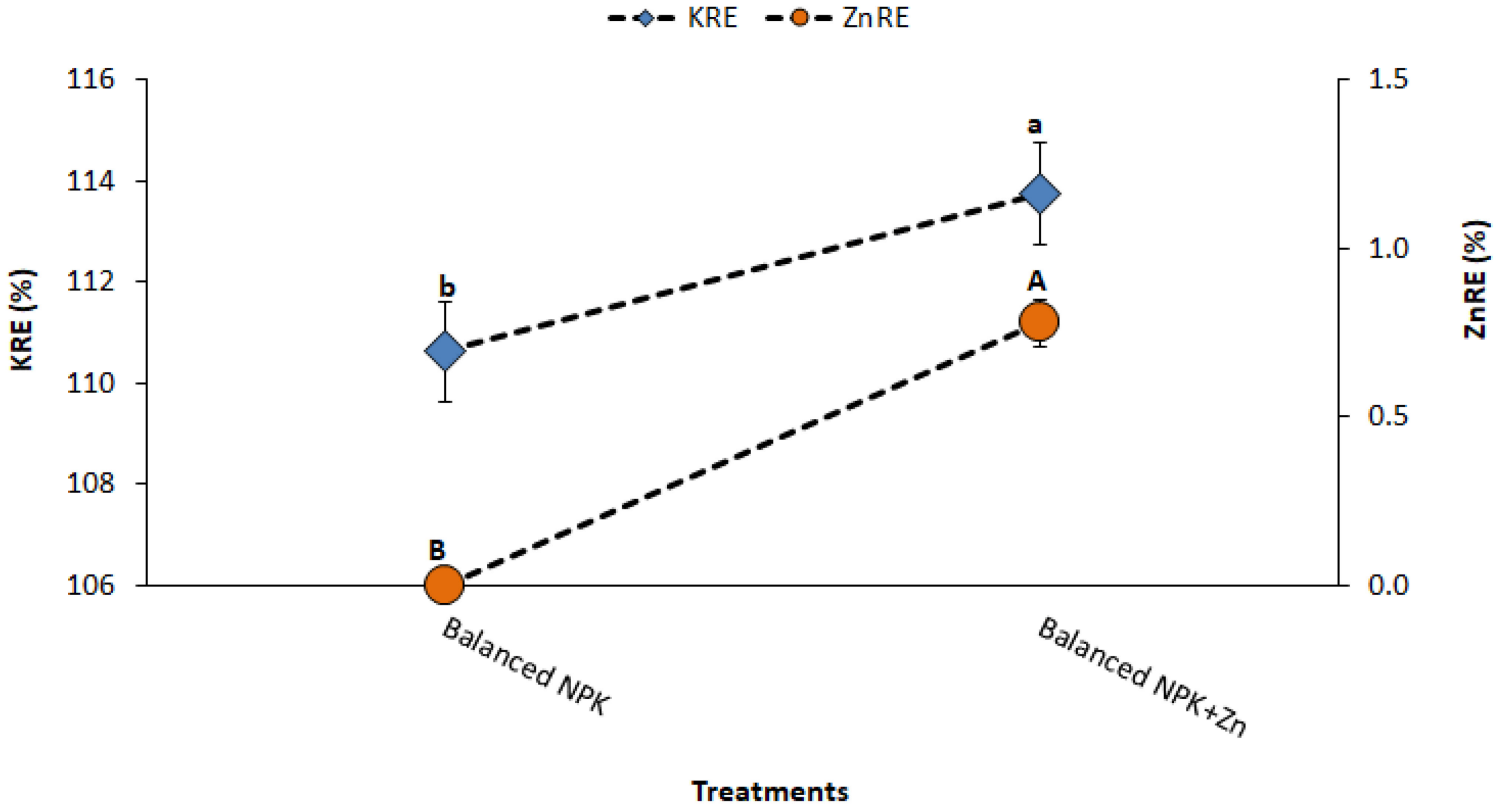
Effect of treatments on KRE and ZnRE. The data is presented as mean ± SD. The letter shows the statistical differences.

**Figure 7:**
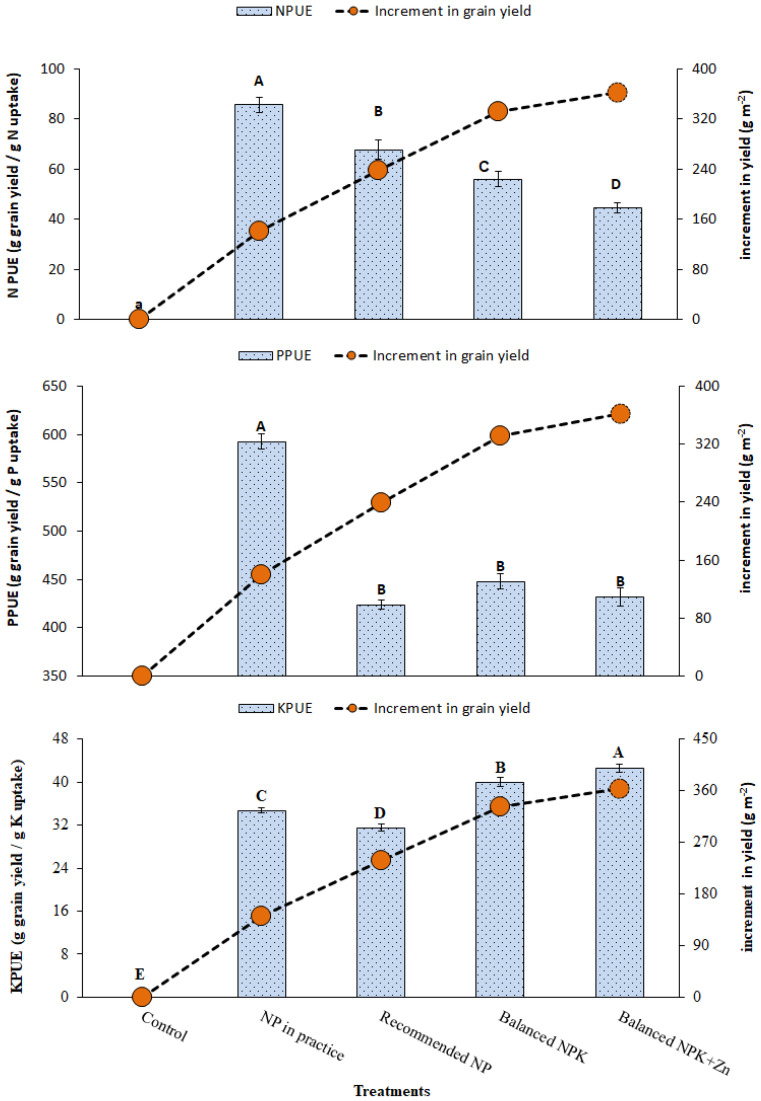
Effect of treatments on NPUE, PPUE, KPUE and increment in yield (g m-2). The data is presented as Mean ± SD. The letter shows the statistical differences.

Application of Zn and K (T_4_ and T_5_) improved the fertilizer use efficiency as compared to the recommended NP nutrition without K and Zn (T_3_). It was found that the combined application of NP, K, and Zn (T_5_) improved the recovery efficiency of applied nutrients, i.e., NRE by 230%, PRE by 136%, KRE by 135%, and ZnRE by 136% as compared to NP-alone application. The results also explore that the combined use of NP, K, and Zn (T_5_) significantly improved the agronomic use efficiency of applied fertilizers such as KAUE by 71%, PAUE by 72%, NAUE by 70%, and ZnAUE by 72% as compared to NP-alone application at the recommended levels (T_3_; **Figures 5**, **6**). It was also noted that both K and Zn played a significant role in lowering the physiological use efficiency (PUE), which means higher yield ratios from the minimum utilization of nutrient uptake or efficient use of nutrients absorbed by plants for grain production. The results showed that NPUE, PPUE, KPUE, and ZnPUE were reduced by 5%, 3%, 3%, and 5%, respectively, as compared to NP-alone application (**Figure 7**). Balancing the N/P/K ratio by increasing the input of K and Zn fertilizers is a practical way to increase the agronomic efficiency.

## 4 Discussion

The results revealed that SPAD chlorophyll contents were improved with the use of integrated nutrient fertilizer strategy. It might be associated with the improved relative water content (RWC) and osmotic potential (Fanaei et al., 2009). Application of Zn also improved the chlorophyll content in maize (Raza et al., 2021), soybean (Weisany et al., 2012), and wheat (Sharma et al., 2005). In addition, photosynthetic rates, transpiration rates, and stomatal conductance were also improved possibly through the involvement in guard cell movement and osmoregulation that ultimately enhanced the stomatal conductance (Imtiaz et al., 2023). K application has also been reported to enhance the gas exchange in plants as well as opening and closing of stomata, especially in cauliflower (Sharma et al., 1994) and maize (Raza et al., 2021).

Improvement in crop physiological processes also leads to an increase in plant height, growth, and performance. The combined application of multi-nutrient fertilizers increased the agronomic as well as yield parameters of the crop. It might be linked with the role of Zn and K in plant metabolic activities and protein synthesis, which also promote plant growth and yield (Ali Khan et al., 2018).

Zn plays a vital role in the plant enzyme known as carbonic anhydrase, which is present in both the chloroplast and cytoplasm, and higher Zn levels increase its activity which, in turn, facilitates the transfer of CO_2_ from the cell’s liquid phase to the chloroplast during photosynthesis and enhances stomatal conductance as well as gaseous exchange in plants (Ahmed et al., 2022). Moreover, the improvements in WUE might be due to the adequate K supply that ensures the osmotic potential regulation, increases the water uptake capability, and ultimately improves the crop yield and water use efficiency (Salem et al., 2022). These findings were observed by Zia et al. (2007) and Johnn (2020).

Furthermore, grain Zn uptake was also improved, and it might be due to the exogenous Zn application that increased the Zn concentration in the soil solution, facilitating its uptake through the diffusion process and a higher share of post-anthesis root (Raza et al., 2021). Application of Zn and K also improves the plant root system, thus increasing the plant’s nutrient uptake efficiency (Jat et al., 2013). Plant K uptake in grain was increased when integrated with Zn. Similar results were reported when Zn and K were applied to maize (Raza et al., 2021). The application of K and Zn at clay exchange sites is replaced and enhances the Zn uptake in plants (Gupta et al., 2018).

Intensive agricultural practices combined with imbalanced fertilizer application have led to the depletion of K and Zn in soil across extensive areas of Pakistan. The scarcity of K in crop production typically arises due to an excessive focus on N and phosphorus fertilization while neglecting K fertilization. Our findings indicate that applying K fertilizer would significantly alleviate the current soil K depletion and, in turn, improve wheat yield. Both K and Zn yielded positive outcomes in terms of photosynthesis rates, transpiration rates, and stomatal conductance along with water use efficiency, advocating the involvement of K in osmoregulation as delineated by Saudy et al. (2023). However, farmers cultivating wheat must consider the application of Zn to ensure sustainable crop production, as the application of Zn improves not only the yield through improved tillering and physiology but also the grain Zn contents.

We conclude that the combination of essential nutrients, especially N, P, K, and Zn, might improve the wheat crop performance and yield grown in nutrient-deficient soils of Pakistan. It is an excellent approach to reaching high food demand. However, long-term studies in different environments and soil conditions need to be studied. The impact of fertilization on soil properties, adopting reduced fertilization technologies, choosing different application methods, enhancing the quality of wheat grain, and economic analysis is an open question.

## 5 Conclusion

We conclude that the application of K and Zn with NP showed maximum improvement in yield attributes, nutrient uptake, and nutrient use efficiency compared to NP-alone fertilization. When NP was applied at the recommended rate, it significantly improved the yield parameters. Still the absence of K adversely impacted the crop yield, nutrient uptake, and fertilizer use efficiency, probably due to the root cause of the poor yield of wheat and nutrient losses on calcareous soil in Multan. As per our hypothesis, K and Zn fertilization can mitigate this yield gap. Our findings recommend that K and Zn should be made an essential part of wheat nutrition management along with NP fertilizers for a higher yield and better quality of produce. Moreover, we do also suggested to conduct long-term trials (10–15 years) to identify the long-term application effects on soil properties.

## Data availability statement

The original contributions presented in the study are included in the article/supplementary material. Further inquiries can be directed to the corresponding authors.

## Author contributions

AN: Formal analysis, Investigation, Writing – original draft. WA: Conceptualization, Project administration, Supervision, Writing – review & editing. TH: Data curation, Resources, Validation, Writing – review & editing. RI: Data curation, Software, Writing – review & editing. SSHS: Conceptualization, Methodology, Project administration, Writing – review & editing. MK: Data curation, Methodology, Visualization, Writing – review & editing. MB: Formal analysis, Software, Writing – review & editing. MR: Conceptualization, Funding acquisition, Writing – review & editing. SS: Data curation, Validation, Writing – review & editing. MA: Conceptualization, Project administration, Supervision, Writing – review & editing. IA: Conceptualization, Investigation, Methodology, Writing – review & editing. AR: Methodology, Validation, Writing – review & editing. RL: Funding acquisition, Writing – review & editing.
